# Shielding Flowers Developing under Stress: Translating Theory to Field Application

**DOI:** 10.3390/plants3030304

**Published:** 2014-07-11

**Authors:** Noam Chayut, Shiri Sobol, Nahum Nave, Alon Samach

**Affiliations:** The Institute for Plant Sciences and Genetics in Agriculture, The Robert H. Smith Faculty of Agriculture, Food And Environment, The Hebrew University of Jerusalem, Rehovot 76100, Israel; E-Mails: noamchayut@gmail.com (N.C.); shiri.naft@gmail.com (S.S.); nahum@dalton-winery.com (N.N.)

**Keywords:** flower development, flowering time, off-season flowering, *Passiflora edulis*, cytokinins, gibberellins, temperature

## Abstract

Developing reproductive organs within a flower are sensitive to environmental stress. A higher incidence of environmental stress during this stage of a crop plants’ developmental cycle will lead to major breaches in food security. Clearly, we need to understand this sensitivity and try and overcome it, by agricultural practices and/or the breeding of more tolerant cultivars. Although passion fruit vines initiate flowers all year round, flower primordia abort during warm summers. This restricts the season of fruit production in regions with warm summers. Previously, using controlled chambers, stages in flower development that are sensitive to heat were identified. Based on genetic analysis and physiological experiments in controlled environments, gibberellin activity appeared to be a possible point of horticultural intervention. Here, we aimed to shield flowers of a commercial cultivar from end of summer conditions, thus allowing fruit production in new seasons. We conducted experiments over three years in different settings, and our findings consistently show that a single application of an inhibitor of gibberellin biosynthesis to vines in mid-August can cause precocious flowering of ~2–4 weeks, leading to earlier fruit production of ~1 month. In this case, knowledge obtained on phenology, environmental constraints and genetic variation, allowed us to reach a practical solution.

## 1. Introduction

Flowers, containing a plant’s reproductive organs, are sensitive to hot ambient temperatures (HAT). The temperature that will actually cause irreparable damage to these organs depends on the species and the genotypes within. Most perennial species reach anthesis once a year, in the spring. Flower initiation and early stages of flower development may occur in the previous summer (apples) or towards the end of winter (citrus) [1]. Assuming that the highest ambient temperatures are reached in summer, HAT will affect initial stages of flower development in species that initiate flowers in summer. In species that initiate flowers after winter, HAT becomes a more serious threat at further stages in reproductive development (pollen viability, stigma receptivity) [2,3,4] or during fruitlet initial development [5]. 

Climate change may likely lead to even warmer temperatures at early stages of flower development in species with summer initiation and in species with late winter initiation. Thus, understanding the damage to reproductive organs caused by HAT and finding means to protect crops from HAT damage is of high importance [6]. Many botanical studies focus on describing and understanding processes, while other applied studies are more focused on providing agricultural solutions. Here, we provide an example of translational research, in which insights from basic studies on *Passiflora edulis* [7] were useful for developing field applications capable of protecting flowers during HAT, leading to out of season passion fruit.

*Passiflora edulis* is a woody perennial vine, originating in Brazil and neighboring countries. The species has quite strong variation in many traits, such as fruit color, self-incompatibility and resistance to climate extremes. The literature traditionally mentions two major edible groups (varieties or forma), the purple (*P. edulis* Sims var. *edulis*) and yellow (*P. edulis* Sims f. *flavicarpa* O. Deg.) passion fruit. Each group has been assigned certain traits and may have originated from different climates, and some hybrids have combined positive agricultural traits [8]. Since traits seem to segregate independently of each other, so that yellow-colored passion-fruit found today in the market might have many traits traditionally assigned to purple passion fruit, it seems simpler to define them all under the name *P. edulis* [9].

Cultivation outside of the region of origin likely began in Hawaii in the 1880s. The first published reports on the cultivation of *P. edulis* came in the 1930s [10,11]. In 2007, world production was estimated at 0.64 Million tons per annum [12], yet it is likely much higher, since most of the production is by small scale farmers for local consumption. In Brazil, the largest producer, the main commercial growth is of *flavicarpa* or its hybrids, producing yellow, relatively sour (high levels of acid), quite large fruit for the juice industry, locally named “Maracujá-Amarelo”. Brazil, Colombia and Ecuador produced 70% of the world production, yet this commodity is rapidly increasing in climates, ranging from cool subtropical to warm tropical (Asia, Africa, Australia, North and Central America, as well as other South American countries). The main product is juice concentrate with the EU consuming 85% [12]; the second product is fresh fruit, for example the purple “Passion dream” (PD) cultivar grown in Israel [13]. 

Flower induction in passion fruit is independent of environment. All non-juvenile nodes contain a leaf, a tendril meristem that produces both a tendril and a flower [14,15] and a vegetative lateral meristem. In apices of healthy plants of the PD cultivar, a new node forms approximately every two days [7]. The rate of growth of the flower primordium and the transition between stages of development along the length of the shoot is more or less uniform. Thus, flowers reach similar sizes and developmental stages at specific, more or less conserved node positions, relative to the apex [7]. Flower primordium formation occurs at node Position Number 6 (P6), meaning that there are five younger leaf primordia till the apex. Sepals are formed at P9, and all four whorls are present at P13. Anthesis, lasting ~12 h, occurs when primordia reach P24–P31 (28.6 nodes on average), ~45 days after the flower primordium has formed. 

Successful flower development requires certain environmental conditions [7,15,16]. Flower primordia of most varieties abort at an early stage of development if exposed to photoperiods shorter than 11 h [15,17]. In many species, photoperiod influences flower induction [18]. In passion fruit, long days (LDs) are necessary for intact flower development and not for flower induction. In farming with no irrigation, a dry season is likely to cause a reduction in bloom [19,20,21]. An additional restraint seems to be low irradiance [22]. 

Primordia of most *P. edulis* varieties are sensitive to hot ambient temperatures (HAT) and abort during warm summers typical for many growing regions, such as Japan, Hawaii, Florida, Southern Australia and Israel [7,11,16,21,23,24,25,26]. In Israel, flowers of the PD cultivar reach anthesis only during spring and autumn and abort during winter, due to short photoperiods [15], and summer (mid-June till October), due to HAT conditions [7]. Flowers reaching anthesis normally set fruit if pollinated, even under HAT conditions. In the field, during summer, PD flowers abort before reaching P17 [7]. During September, daytime field temperatures are normally still high, yet nights become slightly cooler, as measured by hours below 22 °C [7]. PD flower primordia growing during this transition period are exposed to decreasing inhibitory HAT conditions, and the first ones to survive end of summer conditions and reach anthesis do so in the first weeks of October. We identified a genotype, “Anthesis all year” (AAY), capable of developing flowers under summer HAT conditions, yet of no commercial value. In comparison to PD, AAY leaves apparently contain much higher levels of cytokinin [7]. Spraying net house-grown PD plants with a synthetic cytokinin (forchlorfenuron; FCF; commercial name “Guliver”) towards the end of summer (August 25) significantly increased the survival rate of primordia reaching P12–P14 on September 20, enabling them to reach anthesis; yet, this had only a subtle influence on the time of flowering [7]. 

An understanding of the phenology of PD flower development allows us to predict the developmental state of a floral primordium at a certain node position at different time points in the past or future. We can subject plants to changing controlled environments and determine at which developmental stage flower primordia are sensitive to HAT conditions and when they become resistant. 

Under controlled HAT conditions (a 34/22 °C day/night temperature regime), flowers aborted after making stamens and carpels. When PD plants were moved from optimal conditions for flowering (OCF; 22/16 °C LDs) to controlled HAT conditions, primordia that already reached P17 at day of transfer/treatment (DOT) managed to properly reach anthesis. This suggests that primordia reaching P17 are no longer sensitive to this HAT treatment. When PD plants were moved from controlled HAT conditions to OCF conditions, primordia that passed P11 on DOT could not be saved and aborted before anthesis [7]. This suggests that damage caused by HAT is irreversible after primordia passed P11. Pre-treating PD plants with the gibberellin biosynthesis inhibitor, uniconazole (Uni), before transferring them from controlled HAT conditions to controlled OCF conditions, delayed the irreversible damage caused by HAT to primordia passed P14 [7]. It could be that the ability of AAY vines to flower in summer is at least partially due to higher cytokinin levels, which were shown in several systems to reduce gibberellin activity. This might explain why directly reducing gibberellin levels with Uni achieved a more efficient means to protect flowers under controlled HAT conditions. 

### Aim of This Manuscript

Currently, the local industry cannot produce ripe PD fruit between September and late December, because no flowers bloom between June and October. The information we recently gained on the phenology of passion fruit, the stages that are HAT sensitive and the effect of UNI under controlled conditions [7], prompted us in this study to try and translate our basic research to a field application. The heterogeneity in flower development within a vine at any specific time, together with the gradual changes in the environment, provide both a challenge and an opportunity to design practical interventions that cause out of season fruit production in passion fruit. Here, we hypothesized that applications of Uni with or without FCF at specific dates would effectively create a “shield” for flowers developing outdoors towards the end of summer, as temperatures gradually decrease towards the autumn. Here, we conducted a series of field trial experiments over a period of three years and found a treatment that consistently shielded flowers, allowing out of season fruit production. 

## 2. Results and Discussion

**Figure 1:**
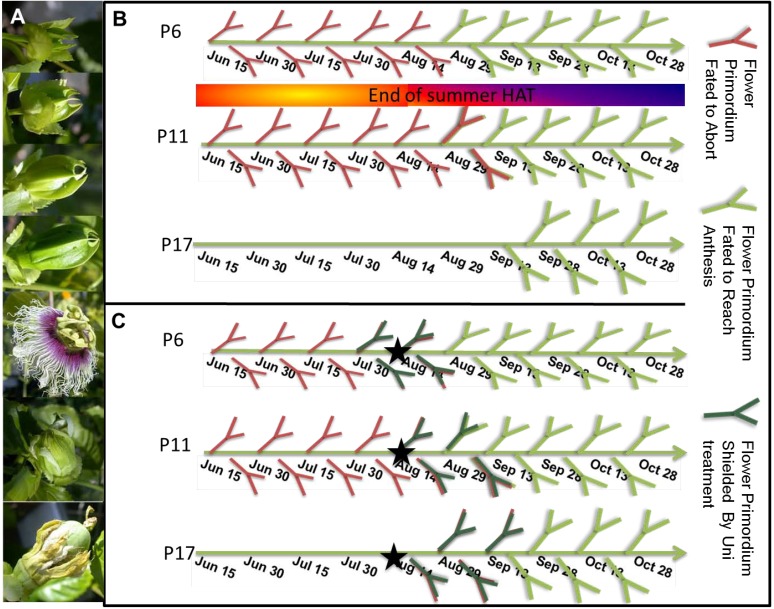
Mapping the fate of flower primordia. (**A**) Pictures of developing Passion dream (PD) flowers past Position Number 17 (P17) under optimal conditions, including the fruit set and the start of fruit development. For earlier stages, see [7]. Anthesis lasts around 12 h; (**B**) The fate of flower primordia during summer and fall. The upper row describes the fate of primordia that reach P6 (initiation of the flower primordium) at different dates. Primordia in red will abort before anthesis, while primordia in green will continue developing till anthesis. The first primordia that will complete development are at P6 on approximately August 28. The middle row describes the fate of primordia that reach P11 at different dates. The first primordia that will not abort (green, P6 on August 28) are at P11 on approximately September 9. The bottom row follows the fate of primordia that reach P17 on different dates. PD primordia do not reach this stage during summer. Normally, a primordium that reaches P17 intact will continue developing till anthesis, no matter the external conditions [7]; (**C**) The fate of flower primordia on vines treated with Uni on August 13 (black star). Similar to (**B**), the upper row describes the fate of primordia that reach P6 on different dates. Primordia in dark green are those saved by the treatment. Due to the treatment, the first primordia that will complete development are at P6 on approximately July 30. Not all of these primordia managed to reach anthesis. The treatment saved primordia that were not older than P11–P13 on day of transfer/treatment (DOT). The first treated primordia that reached P17 likely did so by August 21.

In general, under local Rehovot (32°N) conditions (Supplementary Figure S1), fruit of PD vines ripen in the summer (July–August) and winter (January–March) [7]. In 2009, the first wave of ripe winter fruit (the last week of December to the beginning of January) was set around October 12 (October 5–20), when flowers reached anthesis. For these flowers, using the parameters introduced above [7], we calculated the time in which earlier stages of development were reached. These flowers were initiated (P6) ~45 days earlier on approximately August 28, passed the critical P11 stage on approximately September 9 and the P17 stage on approximately September 21 (Figure 1A,B), when temperatures began to decrease (Supplementary Figures S1 and S2). Between the previous spring and September 9, all flowers that reached P11 aborted due to summer HAT conditions. Uni treatment was shown to shield HAT-subjected PD flower primordia, if they are moved by P14 to OCF conditions [7]. We hypothesized that by applying Uni in the field, we could protect additional primordia; those that already reached P12–14 by September 9 and without Uni normally abort. This should lead to earlier flowering in the field and an earlier yield. To accomplish this, we decided to expose such (P14 on September 9) primordia to Uni from the time their node is formed (P0), which is ~28 days earlier, on August 13 (Figure 1C).

### 2.1. Summer 2009 Net House Trial

**Figure 2:**
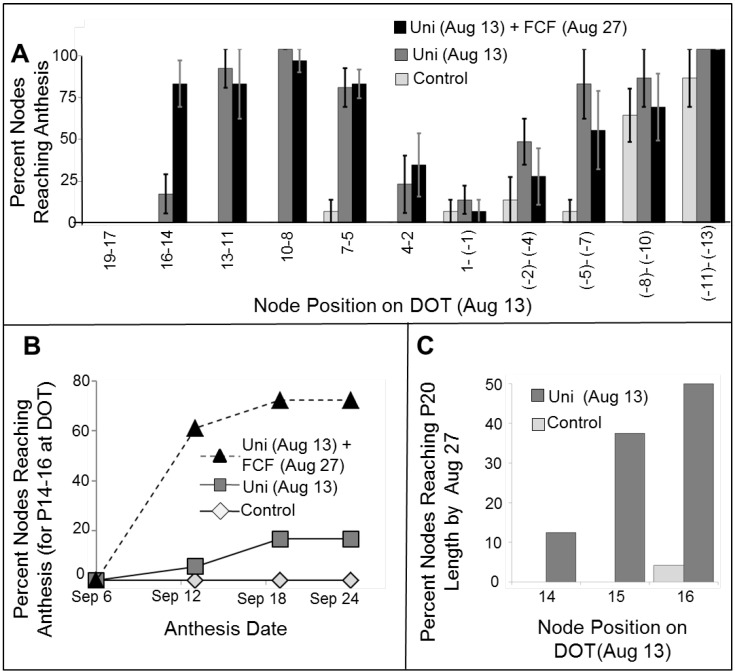
Uniconazole (Uni) and forchlorfenuron (FCF) application on one-branched pot-grown PD plants (2009). The plants were grown under local end of summer conditions, in a net house (see Section 3.3). Treatments: plants sprayed with 200 ppm Uni on August 13 (DOT) or plants sprayed with 200 ppm Uni on August 13 and with 10 ppm FCF on August 25. Control plants were sprayed with water plus surfactant at DOT. Each treatment contained 5–6 plants. (**A**) Percent flower primordia reaching anthesis, per node position on DOT; bars represent the standard error of the mean. Node positions depicted as negative were not formed yet on DOT; (**B**) Anthesis date of P14–P16 (position on DOT) primordia. Control plants did not have developed flowers at these positions, and the first anthesis in control plants occurred on a later date (October 10). The additional FCF treatment caused a higher rate of flowers reaching anthesis at these positions, on an earlier date. For each treatment, the fate of 18 nodes (three nodes in six plants) was followed, and the percent of nodes reaching anthesis was calculated; (**C**) The short-term fate of nodes that were at P14–P16 on DOT, measured two weeks later (August 27). We measured the percent of primordia reaching a length of more than 24 mm, corresponding to normal primordia at P20. Reaching a length similar to P20 is not proof that the primordium is intact. *n* = 18 (one node in 18 plants).

Our initial experiments outside of controlled growth rooms were performed in a net house (see Section 3.3 for the conditions) towards the end of summer. Potted plants were entrained under these conditions from the end of July. Similar to field conditions, the first control (untreated plants) flowers reached anthesis on October 5, and all control plants reached anthesis by October 25. We tested the effects of 200 ppm Uni (DOT: August 13) with or without FCF (10 ppm; applied on August 25). Since we marked node positions on DOT, we could follow and calculate the success rate (primordia reaching anthesis) per node position (on DOT), per treatment (Figure 2A). While we were expecting treated plants to reach anthesis ~1 week (3 nodes × 2 days per node) earlier than control plants, flowers from Uni-treated plants first reached anthesis already on approximately September 10, approximately one month before control plants. The first primordia of control plants that ended up reaching anthesis were at P-3 (position minus 3) on DOT. These primordia reached anthesis in the Uni-treated plants, as well. In addition, primordia that were already formed on DOT, but had not reached P14 (P13–P5), also reached anthesis due to the Uni treatment (Figure 1C and Figure 2A). This might suggest that the shielding ability of Uni in the field is on existing primordia (not younger than P5–P6). Our field results also suggest that Uni shielding is much stronger in the end of summer conditions compared to the previously tested simulation of HAT under controlled conditions [7]. 

When the Uni treatment was followed by FCF 12 days later, the success rate of additional primordia, those at P14–P16 on DOT, increased (Figure 2A). We followed the fate of these particular primordia (P14–P16 on DOT). We recorded when these primordia reached anthesis (Figure 2B). Obviously, those of control plants aborted before reaching anthesis. The addition of FCF allowed most of these primordia to reach anthesis by September 12 (Figure 2B). An FCF treatment without previous Uni treatment did not protect these flowers [7]. This suggests that the early Uni treatment primed these primordia to respond to the FCF treatment, yet it was not sufficient, by itself, to shield these primordia till anthesis. We tried taking a closer look at these P14–P16 primordia, to better understand their fate (primed, but not capable of reaching anthesis) in Uni-treated plants. Although they did not reach anthesis, their size by August 27 in Uni-treated plants was much larger than untreated plants (Figure 2C). 

In this experiment, there was a clear advantage to the additional FCF treatment. For farmers, considering the cost (chemicals, manpower) of this extra spray, the extra gain of 2–3 additional primordia is likely not worth the effort. In the field experiments described below, adding FCF to Uni never provided a substantial difference in the time of yield, so further experiments described here focus on the application of Uni alone.

### 2.2. Summers of 2010–2011 Net House Trials

**Figure 3:**
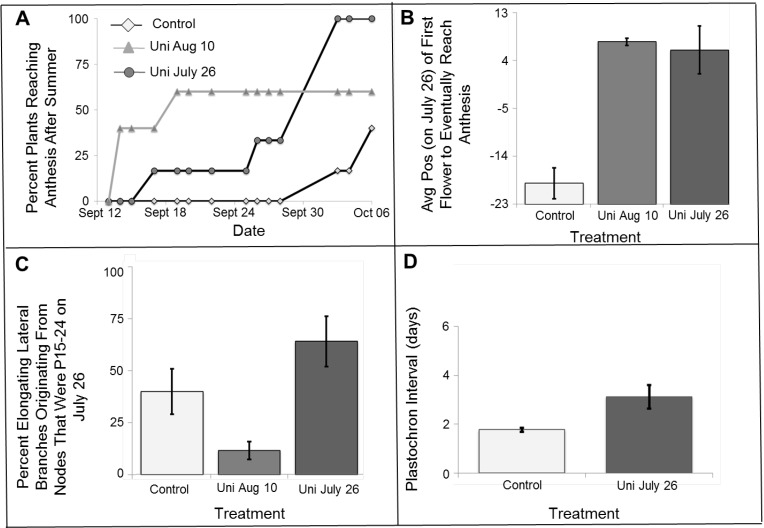
Uni application on one-branched pot-grown PD plants (2010). The plants were grown under local end of summer conditions, in a net house (see Section 3.3). Treatments: plants sprayed with 200 ppm Uni on July 26 (DOT) or on August 10. Control plants were sprayed with water plus surfactant at DOT. Six plants were analyzed for each treatment. (**A**) The percent of plants reaching anthesis from September 12 (when treated plants reached anthesis) until October 6 (when control plants started reaching anthesis); (**B**) Average relative position on DOT (along the main branch) in which sequential primordia (more than one flower) reached a blooming stage. For control plants, these were primordia that were not yet formed (negative value) at DOT; (**C**) The effect of Uni treatments on the elongation of lateral meristems from nodes that were positioned at P15–P24 on DOT. The measurement was performed on August 19; (**D**) The effect of Uni on July 16 on the plastochron interval (see Section 3.4). For each plant, we counted the number of newly-formed nodes during the experiment and divided the experiment duration in days by the node number. Bars in (**B–D**) represent the standard error of the mean.

In the summer of 2010, we conducted a similar experiment using the same net house. First, we wanted to repeat the experiment, testing whether the Uni treatment will be effective again. In addition, we asked whether we could reach an even earlier date of anthesis by applying Uni earlier in the summer. Two hundred parts per million of Uni were applied on July 26 or August 10. Control plants (water plus surfactant sprayed at DOT) began anthesis on October 3, while treated plants (both Uni treatments) reached anthesis ~20 days earlier (Figure 3A). By October 3, all plants treated with Uni on July 26 reached anthesis, while in control plants, only 17% reached anthesis by this date (Figure 3A). The first fruit from treated plants ripened on December 16, more than two weeks before fruit from control plants. 

The first control flowers reaching anthesis after summer were, on average, at ~P-(minus)19 on July 26 (Figure 3B). The first intact August 10 Uni-treated flowers reaching anthesis were, on average, at ~P7.5 on July 26. Thus, Uni treatment in mid-August clearly shielded flowers one year after another. Spraying plants with Uni at an earlier date (July 26) did not shield extra flowers (Figure 3B). Thus, this specific Uni treatment, when provided at an earlier date, was not sufficient to enable flowers to reach anthesis before September under local conditions. It seems that our original calculated “window of intervention” on approximately August 12 was appropriate. 

Uniconazole, as an inhibitor of gibberellin biosynthesis, can cause growth retardation in plants, including passion fruit [27]. In this experiment, we also followed the elongation of lateral branches in response to the treatment. Nine days after treatment, the elongation of lateral branches was inhibited compared to control plants, yet 24 days after treatment, elongation was restored (Figure 3C). 

While the Uni treatment shielded flowers, the July 26 treatment also increased plastochron intervals by ~1.7-fold (Figure 3D), in other words, slowing down the production of new primordia and possibly flower development. This likely explains why a Uni-treated flower that reached P7.5 on July 26 required an additional 50 days to reach anthesis (September 14). It appears that the full agricultural potential of Uni shielding of primordia from HAT is partially masked by slower flower development. This effect of Uni on plastochron intervals may depend on environmental conditions at the time of application, since it was not noticed in other Uni treatments.

In the summer of 2011, we performed an additional one-branched potted PD plant trial in the same net house. Treatments were 200 ppm Uni on either July 28 or August 11 compared to untreated plants (control). In control plants, the first flowers appeared on October 7, and the average date of first anthesis was October 24. In this trial, an earlier date of application (end of July) led to an even earlier date of anthesis (Supplementary Figure S3). Uni treatment on July 28 caused first anthesis, in the beginning of September, while Uni treatment on August 11 caused early (less dramatic) flowering, on around September 21. This difference between years could be due to a cooler period before August 11 in the summer of 2011, compared to 2010 (Supplementary Figure S2). 

### 2.3. Summer 2010 Experimental Field Trial

**Figure 4:**
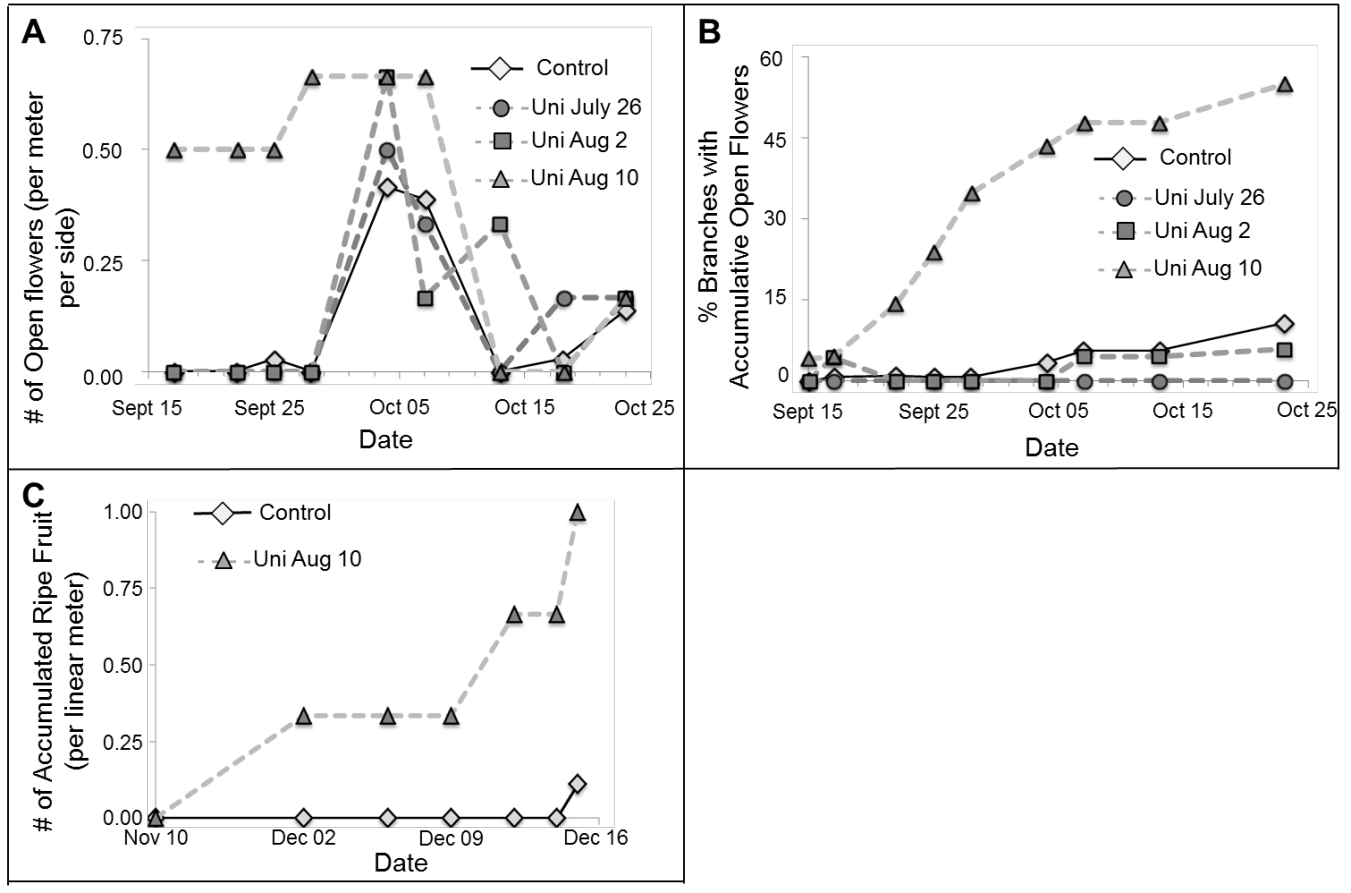
Uni spray on PD vines in an experimental field (2010). Treatments: 200 ppm Uni sprayed on July 26/August 2/August 10 and control (not treated). We visited the plot seven times in a period of 22 days starting September 15. (**A**) The number of open flowers per running meter of trellis per side on that certain day; (**B**) For each treatment, we gathered information on 20 random shoots (10 per side, west or east) that were appointed and marked before treatments began. The accumulative percentage of blooming branches is presented; (**C**) Accumulated ripe fruit number per linear meter of trellised vines. Fruit was defined as ripe upon abscission. We collected and counted the number of fruit on the ground on each visit.

In the summer of 2010, we also tested the effect of Uni treatment at different dates (July 26, August 2 and August 10) on flowering in the faculty experimental orchard (no nets, field conditions). We conducted a “snapshot” survey by counting the number of open flowers per trellis running meter at a certain time point, every few days (Figure 4A). In this count, some of the flowers reaching anthesis are likely missed, and their distribution among branches is not documented. Thus, we also marked random branches in advance and recorded the date on which the first flower on them reached anthesis. Here, if we missed an open flower, we could still record its bloom a few days later, since it is still on the branch. This allowed us to calculate the accumulation of branches reaching anthesis (Figure 4B). Later on, we counted ripened fruit (fruit that detached) per trellis running meter (Figure 4C). Here, again, we could measure the accumulation of fruit, even when visiting every few days, since the fruit that fell remained on the ground till our next visit. 

The first intact control flowers reached anthesis between October 4 and 7 (Figure 4A). The most effective Uni treatment in causing early flowering in this trial was the latest treatment on August 10. Here, plants at anthesis were detected in the first date of observation, September 17 (Figure 4A). By October 13, ~50% of treated branches reached anthesis, while only ~6% of control branches reached anthesis (Figure 4B). Ripe control fruit began to abscise December 15, 72 days after sighting the first anthesis (Figure 4C). Ripe August 10 Uni-treated fruits began abscising December 2, 76 days after sighting the first anthesis (Figure 4C). By December 19, the treated vines produced nine-fold higher levels of ripe fruit (Figure 4C). 

The two earlier treatments with Uni, on July 26 and August 2, did not cause a significant change in the flowering pattern of the plants, compared to control untreated plants. These treated plants had much shorter internode lengths compared to control plants, as is expected from Uni treatment [15]. Therefore, Uni treatment at earlier times definitely had an effect on plants, but did not cause obvious shielding of flowers. 

### 2.4. Commercial Field Trials 2010–2011

**Figure 5:**
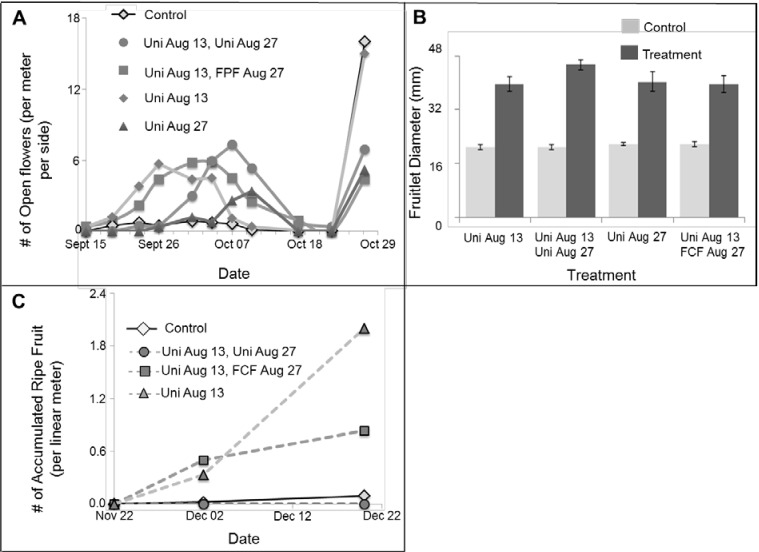
Uni and FCF spray on PD vines in a commercial orchard (2010). Treatments: 200 ppm Uni on August 13/on August 13 and on August 27/200 ppm Uni on August 13 and 10 ppm FCF on August 27/Control (water and surfactant sprayed on August 13). (**A**) Counts of open flowers on several occasions between September 19 and October 27; the latter date is when flowers in the control plants began to reach anthesis. For each date, the average number of flowers reaching anthesis per running meter of trellis per side is presented; (**B**) Fruitlet size (diameter in mms) on November 1: the two largest developing fruits per running meter were documented in each treatment and in the control plants growing in the same orchard line; (**C**) Accumulated number of ripe (abscised) fruits (as in Figure 4C).

In the summer of 2010, we also made our first attempt to shield flowers in a commercial PD orchard (Moranim subtropical fruits LTD; Section 3.3.3). Vines were sprayed with 200 ppm Uni with a hand pump backpack sprayer on August 13, August 27 or on both dates. We also tried adding FCF (August 27) to a single Uni (August 13) treatment to see if adding FCF has a more pronounced affect in the field. Control vines (on each treated row) were sprayed with water and surfactant on August 13. We conducted a “snapshot” survey by counting the number of open flowers per trellis running meter at a certain time point, every few days, between September 15 and October 27. Passion fruit flowers remain open for ~12 h, so every flower was counted only once. Control flowers first reached anthesis at a rate of at least one flower per trellis-meter by October 27 (Figure 5A). The single Uni treatment on August 13 caused the earliest flowering, as flowers reached anthesis at a rate of at least one flower per trellis meter already by September 19. Adding an FCF treatment or a second Uni treatment on August 27 had no added benefit in causing precocious flowering. A single Uni treatment at a later date (August 27) was also less efficient (Figure 5A). It could be that the delay in flowering due to the second treatment of Uni is also because of it causing a slower plastochron interval (not measured).

Fruit of the PD cultivar reached final size ~20 days after fertilization. On November 1, we returned to the orchard to quantify fruitlet size. At this time, fruitlets would have reached their final size (~40 mm in diameter) if they developed from a flower that reached anthesis before October 13. We chose for each trellis meter and side the two largest fruits per treatment. Altogether, the fruit diameter was calculated for 20 fruits per treatment (Figure 5B). The largest control fruits from each row were, on average, 20 mm in diameter. Under ideal conditions, PD fruit reaches this size five days after pollination. Thus, these fruit likely came from flowers that reached anthesis on October 27, when we first observed the first uniform anthesis in control plants (Figure 5A). The largest fruit from the treated plants were all significantly larger, reaching a ~40 mm diameter, suggesting that they developed from flowers that reached anthesis before October 13. Finally, we collected data on initial ripe fruit in the orchard. We visited the orchard four times between November 22 and December 22 and counted the number of accumulated ripe (abscised with full color from the vine) fruit per meter of trellis per treatment (Figure 5C). Not surprisingly, fruit from the treatment that caused early flowering (Uni on August 13) ripened before the control fruit, thus extending the season of yield. The treatment affected ripening date more significantly than it affected anthesis date. One explanation for this may be that in the transition period from September to December, in which local temperatures are slowly declining, fruit maturation takes longer. Thus, the benefit of setting fruit in September *versus* October is that the earlier fruit will be exposed to warmer temperatures and will mature more rapidly. Thus, in this period, earlier anthesis could cause an even more pronounced earlier fruit ripening. A relative delay in fruit ripening was observed in Uni-treated plants that were later treated with FCF (Figure 5C). It is not clear at this point if this effect is significant and repeatable and, if so, what might be the reason for it. 

In the summer of 2011, we conducted additional trials in commercial orchards at two different locations: Moranim LTD and Mr. Isaac Tzipori’s farm (see Section 3.3.3). At the Tzipori farm, three rows of trellised PD vines were left untreated, while three other rows were sprayed with 200 ppm Uni and surfactant on August 10, using a hand pump sprayer. In Moranim LTD commercial orchard trellised PD vines were left untreated or sprayed with 200 ppm Uni and surfactant on August 12 using an air blast sprayer. We visited the farms when notified that control blooming started (Tzipori’s on September 22 and Moranim on September 27). We chose four branches from both sides of the treated and untreated rows (three rows in Tzipori’s and six in Moranim). The branches chosen were those that seemed to have the most flowers and in which growth did not stop (apical meristem still active). We counted the number of flowers that already opened (since the end of summer) on each of the branches. 

**Figure 6:**
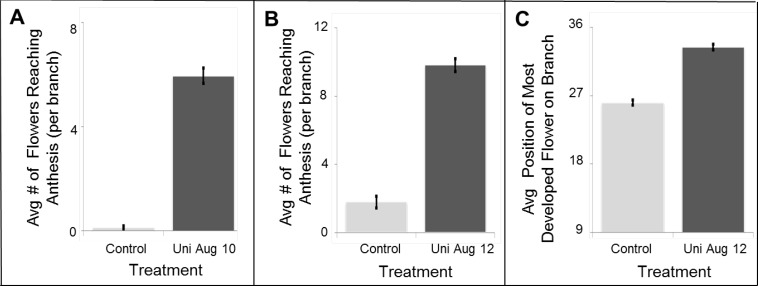
Uni spray on PD vines in commercial orchards (2011). (**A**) Tzipori farm treatments: 200 ppm Uni and surfactant sprayed on 10 August and control untreated plants. The average number of flowers that reached anthesis by September 22 per branch is presented; (**B**) Moranim LTD Treatments: 200 ppm Uni and surfactant sprayed on August 12 and control-untreated plants. The average number of flowers that reached anthesis by September 27 per branch is presented; (**C**) The same experiment as in (**B**), calculating the average relative position from the apex (on September 27) of the first flower reaching anthesis on each branch. A higher number depicts the shielding of additional primordia. Bars in (**A–C**) represent the standard error of the mean.

**Figure 7:**
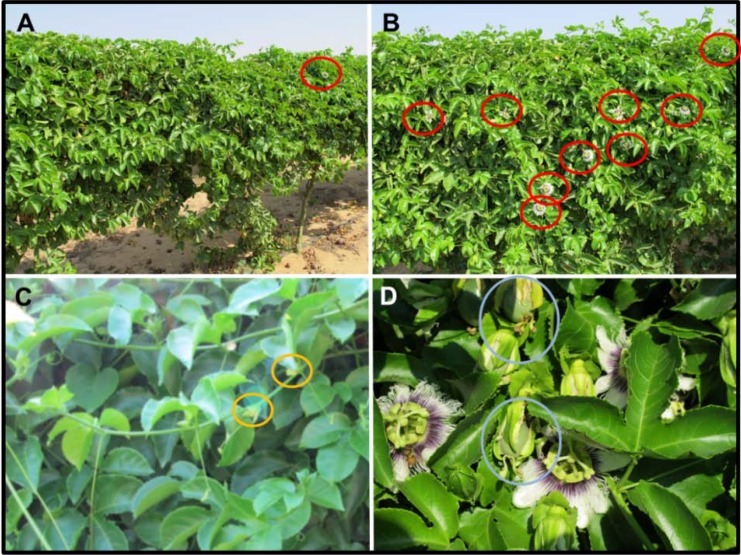
Pictures of PD vines in commercial orchards (2011). (**A**,**B**) Tzipori farm on September 22 with treatment as in Figure 6A. Most control plants (**A**) did not reach anthesis, while treated plants (**B**) are blooming. Red circles mark flowers at anthesis. (**C**,**D**) Moranim LTD farm on September 27 with treatment as in Figure 6B. Aborted flowers (orange circles) can be seen in control vines, while treated vines contain flowers at anthesis attracting pollinators and setting fruit (light blue circles).

In both experiments, spraying 200 ppm Uni on approximately August 10 resulted in a substantially earlier bloom (Figure 6A and Figure 7A,B). In Tzipori’s control, untreated vines, no flowers reached anthesis on an earlier date, while three of the 24 branches contained an open flower on the day of measurement. In contrast, all Uni-treated branches contained previously and currently open flowers, with numbers of flowers per branch ranging from three to eight, with an average of six flowers that had already reached anthesis, per branch (Figure 6A). In Moranim, by September 27, Uni-treated vines contained on average five-fold more flowers that had already reached anthesis, compared to control vines (Figure 6B and Figure 7C,D). We also measured the position of the most developed flower (or fruit) since the end of summer (Figure 6C). In Uni-treated vines, the average position was P33, and in control vines, it was P26. Assuming an average of 28.6 nodes for anthesis, the treated plants potentially had 4–5 set fruit per branch by this date. The presence of developing fruit in treated vines was obvious when studying the vines (Figure 7D).

When conducting a series of field trials meant to overcome an environmental constraint, it is expected to find variation in environmental conditions between years and locations (Supplementary Figure S2). Nevertheless, we were able to repeat our main result in three consecutive years and in four different locations and growth conditions: earlier flowering and fruiting as a result of a single spray of 200 ppm of Uni on approximately August 12. The degree of damage caused to PD flowers by HAT conditions vary with temperature regime (Supplementary Figure S4). The ability of Uni treatment to shield PD flowers from HAT conditions depends on the scale of heat to which flowers are exposed. For example, Uni treatment, although promoting flower development up to a certain stage, did not bring about anthesis under controlled HAT conditions of 34 °C/22 °C day/night regime (Supplementary Figure S4b). Thus, it is expected that the success of earlier (before mid-August) applications of Uni in the field may vary with the conditions in every year, as shown here for the net house trials of 2010 (Figure 3) and 2011 (Supplementary Figure S3). 

### 2.5. Summary

The heterogeneity in flower development within a vine at any specific time, together with the gradual changes in the environment, provide both a challenge and an opportunity to design practical interventions that cause out of season fruit production in passion fruit. Our previous studies suggested that reducing gibberellin levels and increasing cytokinin levels (perhaps causing a reduction in gibberellin activity) might both be potentially useful in protecting flower development under HAT [7]. Here, an understanding of stages in which PD flowers are susceptible to HAT and comprehension of the species plastochron pace allowed us to reach the correct time of intervention, which enabled flower development, leading to out-of-season fruit in commercial field trials. Understanding the dynamics of the primordia development under changing environments helped to define a window in development in which a simple chemical treatment could shield flowers and extend the bloom season and the period in which fruit is available for marketing.

During three consecutive years, in four different locations, we have shown that by spraying PD vines in mid-August with 200 ppm Uni, we can extend the anthesis period by 3–6 weeks, allowing additional flowers to reach anthesis. These flowers can set fruit and ripen much before the untreated fruit, leading to a longer period of fruit production for farmers. In the northern hemisphere, this also allows producing fruit towards the holiday season (December–January), when demand is much higher. This treatment is not costly, suggesting a substantial gain in profit.

Since normal marketable fruits were indeed produced by the Uni-treated flowers, the treatment did not seem to interfere in fertilization, fruit development and maturation. Successful pollination in local orchards depends on honeybees, which are introduced by farmers during bloom, or by hand pollination. Clearly, earlier bloom will require these interventions at an earlier date. 

While we have not tested this treatment in other cultivars or in other growing regions outside of Israel subjected to HAT conditions during summer, we think it is quite likely that it will cause a similar, positive affect. A promising approach will be to breed for cultivars that are less sensitive to environmental constraints and that still have high fruit quality. The local commercial practices are quite intense (irrigation, NPK fertilization, vine replacements every ~5 years). We cannot rule out that under less optimal conditions, other constraints may reduce the shielding effect of the Uni treatment. 

Interestingly, previous reported field trials with gibberellin biosynthesis inhibitors in passion fruit provided no evidence for a change in the time of anthesis [27,28,29]. This could be due to the time of application. Here, we saw that in some of our trials, earlier application of Uni had no effect on flowering. In addition, the time in which the effect of a treatment is measured may affect the results recorded. In most other fruit crops, for example pome fruits, the sequence of events beginning with flower primordia induction until bloom can take ~260 days. In PD, and probably other passion fruit cultivars, anthesis occurs ~45 days after primordia initiation. The Uni treatment affects primordia that are already developing, so that the effect of the treatment on flowering can be seen in a relatively short time (~30 days) and will remain unnoticed if the orchard is revisited after two or more months and only the flowers are monitored. 

Since reducing levels of gibberellin enables further PD flower development under HAT conditions, it appears that gibberellin inhibits flower development under HAT conditions. Gibberellin may be diverting photosynthetic assimilates from developing flowers to other organs, as was suggested in bougainvillea [30]. 

## 3. Experimental

### 3.1. Plant Material

The cv. “Passion dream” (PD) is the most common commercially grown passion fruit in Israel. It was previously described [15] as an F_1_ hybrid of yellow and purple passion fruit. Unlike seed propagated cultivars commonly used in many other countries, the PD highly heterozygous cultivar is propagated by vegetative rooted cutting. Thus, both commercial and experimental orchards vines described in this paper are genetically identical. Plants used for the net house experiments were rooted from cuttings taken from vines of the experimental faculty orchard.

### 3.2. Measuring Size of Flower Primordia

The flower primordium size was determined by measuring the length of bracts, sepals and complete primordium, including pedicel, as was described previously [7,15].

### 3.3. Growth Conditions

In the manuscript, when discussing plants in pots, we use the term “plants”, and when discussing plants in the field, we use the term “vines”. 

#### 3.3.1. Net House

The faculty net house is a cubic white net shed, which reduces light intensity by around 10%, keeps birds and insects away, but maintains the seasonal outdoor temperature and day length conditions. In the summer of 2009, 2010 and 2011, PD plants were grown in 18-cm diameter, well-irrigated pots under net-house conditions at the Faculty of Agriculture. While plants were growing and acclimatizing to the conditions, we pruned emerging secondary branches, allowing only one branch to continue its growth. These plants were termed: “One-branched pot-grown PD plants”. After DOT, we continued the removal of emerging side branches in 2009. In 2010–2011, in order to measure the treatment effect on side-branching, we ceased pruning the plants on DOT. In all experiments, we followed the fate of the developing flowers of the main branch. 

#### 3.3.2. Experimental Field Trial

The Faculty of Agriculture experimental field is in Rehovot, Israel (31°54'12''N; 34°47'47"E). Rooted vegetative cuttings of PD plants were planted in the field in June, 2007. Eight irrigated trellised rows (north to south) of PD vines, each row divided into two (north and south) stretches of 6 meters, were given different treatments 

#### 3.3.3. Commercial Orchards

Moranim Subtropical fruit LTD: trellised irrigated PD plot planted northeast to southwest. Location: 31°52'56''N, 34°50'46"E. The experiments were held in summer 2010 and 2011 at different locations of the farm. 

Isaac Tzipori farm LTD: Location: 31°11'52''N, 34°22'38"E. The experiment was held in summer 2011. 

### 3.4. Calculating Plastochron Intervals at the Shoot Apex of PD

We calculated plastochron intervals [31,32] of successive leaf primordia at the shoot apex of PD. The unfolding of the PD leaf is easily visible and occurs independent of environment, on average, around (~)15 nodes (varies 14–16) from the apex [15]. In other words, if we follow the fate of a leaf primordium formed in the apex, once 14 additional nodes are formed, this leaf will likely unfold. All older nodes will contain open leaves, while all younger nodes contain folded leaves. To calculate the plastochron interval, we marked the main branch twice at the node containing the youngest unfolded leaf and divided the number of days between the two marking time points by the number of newly formed nodes between the two marks. Having a visible marker (leaf unfolding) that can easily tell us at any time the distance (in nodes) from the meristem, together with knowing the plastochron, allowed us to predict for every visible node its current location relative to the apex where it will be, relative to the apex, in the future, and where it was, relative to the apex, in a past date of interest. 

### 3.5. Hormone Treatments

To reduce GA biosynthesis, we used the commercial product “Magic” (Sumitomo Chemicals, Hyogo-Ken, Japan), which is 50 g/L uniconazole (Uni), a triazole capable of inhibiting P450 *ent*-kaurene oxidase (CYP701). The concentration of Uni in spray was 200 ppm. To increase CK levels, we used the commercial product “Guliver” (Jiangsu institute of ecomones, Jiangsu, China), imported by Tarsis Agricultural & Industrial Chemicals Co. Ltd., Petah Tikva, Israel. “Guliver” is an aqueous solution of forchlorfenuron (FCF), a synthetic CK. The applied concentration was 10 g/L. The concentration of FCF in spray was 10 ppm. In all sprays, we added 0.025% Triton X-100 as a surfactant. For net house trials, we used small hand plastic sprayers. In the experimental field trial, we used hand pump backpack sprayers. In the commercial orchards, a hand pump backpack sprayer or an air blast sprayer was used, as stated in the text. Spraying of plants was performed in the early morning.

## 4. Conclusions

Agriculture is in need of constant improvement due to growing human needs and increasing, often man-made, environmental constraints, such as a warmer environment [6]. Many improved agricultural practices originated from a chance set of circumstances leading to a phenomenon noticed by a keen eye, while others were developed from methodical, lengthy trials ending with a winning combination. The use of plant growth regulators to overcome a developmental constraint in plants has provided enormous benefits to agriculture. One common method for identifying the right cocktail is called “spray and pray”. In such a trial, a wide matrix of chemicals, concentrations and schedules is used in an attempt to find a winning combination. This could take a very long time and many resources. While we do not challenge here the added benefit of prayers, our study implies that in some cases, a better comprehension of the underlying developmental events might fast-forward the process. 

## Supplementary Files

Supplementary File 1
