# Supplementary material for: Shielding Flowers Developing under Stress: Translating Theory to Field Application
Source: Plants (Basel). 2014 Jul 11;3(3):304–23. doi: 10.3390/plants3030304 (PMC4844345; doi:10.3390/plants3030304)
Supplement: Supplementary File 1 [file plants-03-00304-s001.pdf]

## Supplemental Material

## Experimental

The controlled-environment glasshouse "Phytotron" experiments described in Supplemental Figure 4 were conducted at the Faculty of Agricultural, Food and Environmental Quality Sciences, Rehovot, Israel. The glass-covered growth rooms transmit ~80% of outside solar radiation. Plants were exposed to Long Days (LDs; 16/8 h light/dark cycles) were maintained by extending the natural day length with supplementary lighting (3–5 μmol/m$^2$s photosynthetically active radiation at the plant level) using 75-W incandescent tungsten bulbs (LM960 Osram GmbH, München, Germany). Different day/night temperature regimes were used: 34/28 °C, 34/22 °C and 28/22 °C. Changes between day and night temperatures were gradual, spanning three hours.

**Figure S1.** (**A**) Diagram based on data from the nearby Beit-Dagan weather station taken from the Israel Meteorological Service http://www.ims.gov.il/IMS/CLIMATE. Data are the average hourly temperatures per month based on data from 1995–2009. The mustard color represents hours in which average temperatures exceed 22 °C, and the dark red color represents hours in which average temperatures exceed 29 °C. The white color represents hours in which average temperatures were 22 °C or below. Notice that such temperatures are normally not reached in July and August. (**B**) Data for the summer of 2009 from an onsite data station placed in the experimental orchard in the Faculty of Agriculture. We measured hours per day in which the temperature was above 29 °C. No data is available for this station in 2010–2011.

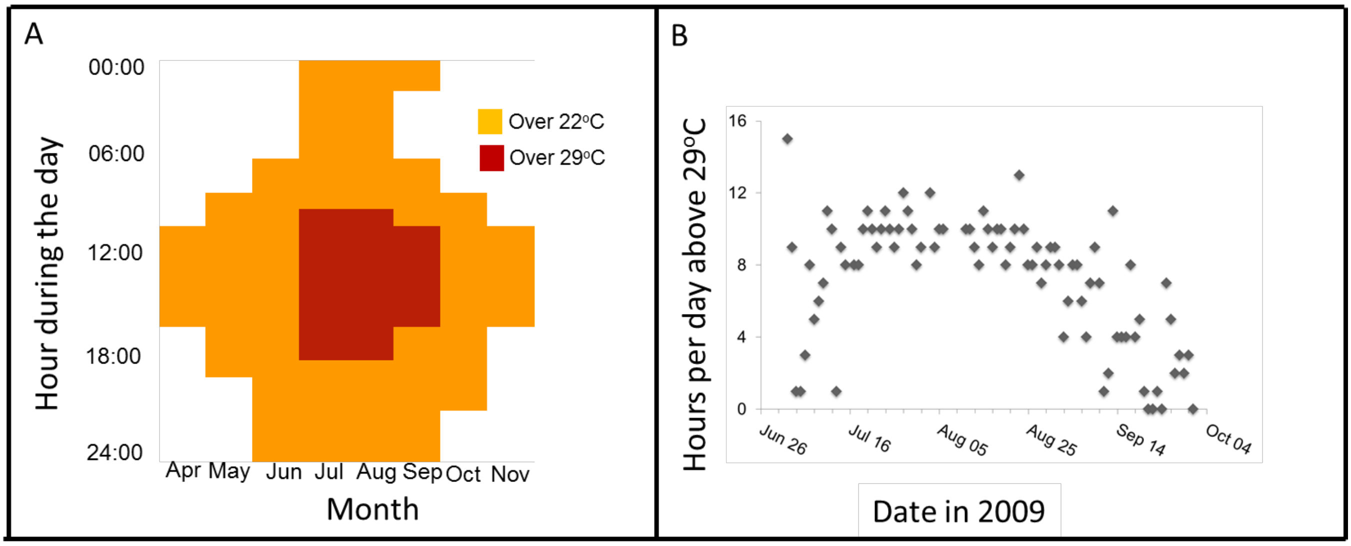

**Figure S2.** Local weather conditions during the summer, Rehovot, Israel. (**A–C**) Maximum (red), minimum (blue) and average (green) daily temperatures from a nearby weather station INESTZIO1 in Kfar Aharon, Israel, from http://www.wunderground.com/personal-weather-station/dashboard?ID=INESTZIO1, during the summers of 2009, 2010 and 2011. The station is missing data on 22 days in August 2010. A horizontal dashed line marks 29 °C.

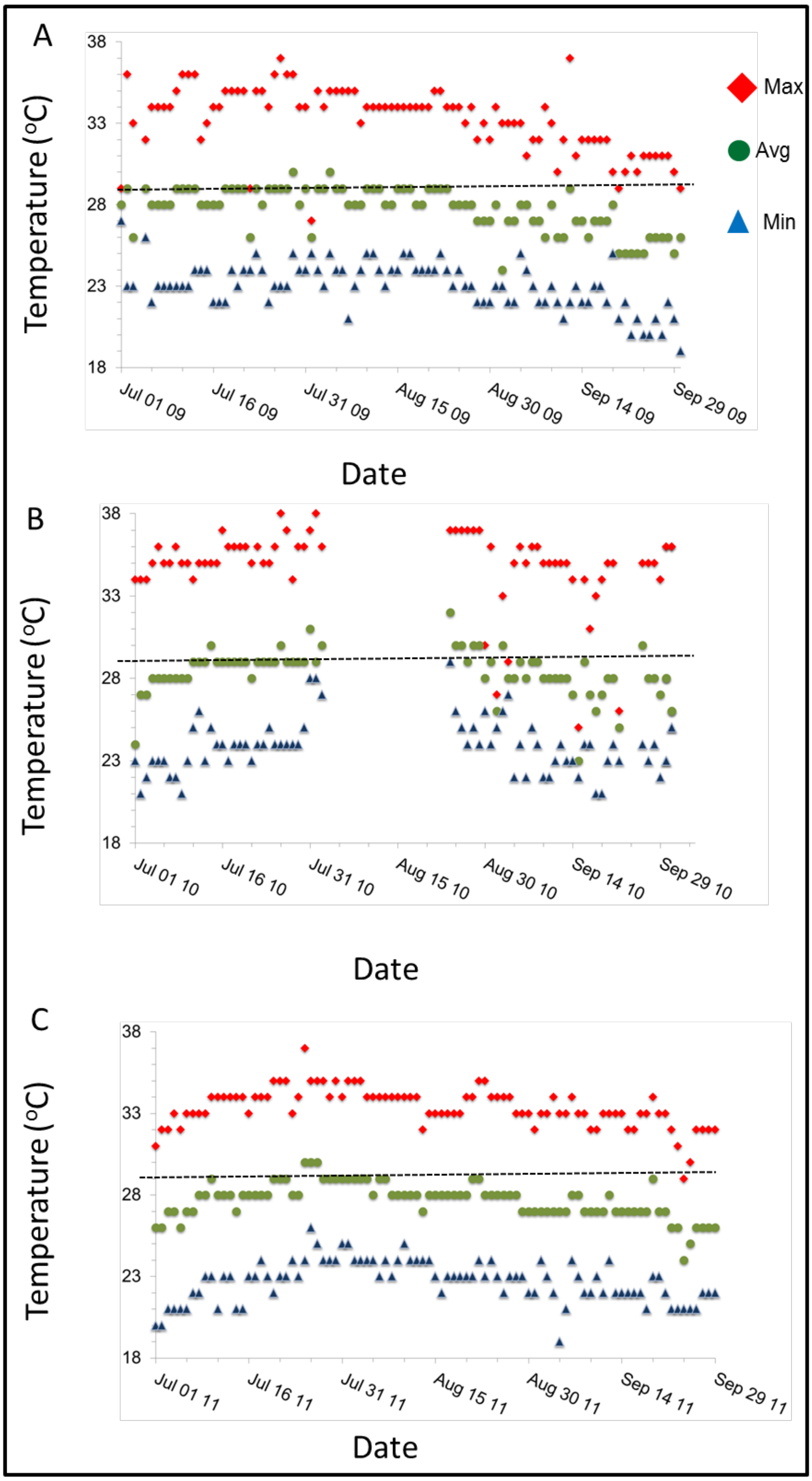

**Figure S3.** Uni application on one-branched pot-grown PD plants (2011). The plants were grown under local end of summer conditions, in a net house (see Section 3.3). Similar to 2010 (Figure 3), secondary branches formed during the summer after DOT were not pruned. Treatments: plants sprayed with 200 ppm Uni on July 28 (DOT) or on August 11. Control plants were sprayed with water plus surfactant on the first DOT. Six plants were analyzed for each treatment. (**A**) Average number of days from July 28 till the first flower reaching anthesis, per plant. (**B**) Average position (on July 28), per plant, of the first flower that reached anthesis. In control plants, the position is negative, since the first primordia that eventually flowered were not formed yet on July 28. Unlike the 2010 net house experiment (Figure 3), an earlier date of application was more effective. Uni treatment on the 28 of July caused first anthesis within ~32 days, in the beginning of September. Uni treatment on August 11 caused early (less dramatic) flowering, on around September 21. Plants treated on DOT1 need 32 days and on DOT2 need 41 days to get flowers that successfully reach anthesis. The first treatment triggers plants to show flowers in anthesis 50 days faster and the second 30 days faster than control flowers. In control untreated plants, the first flower to reach anthesis was at ~P8 on July 28. Uni treatment on this date saved flowers at ~P15 and younger. Uni treatment on August 11 saved flowers that on July 28 were at P6.8 or younger. These primordia were on average at ~P14 on the day they were treated (August 11).

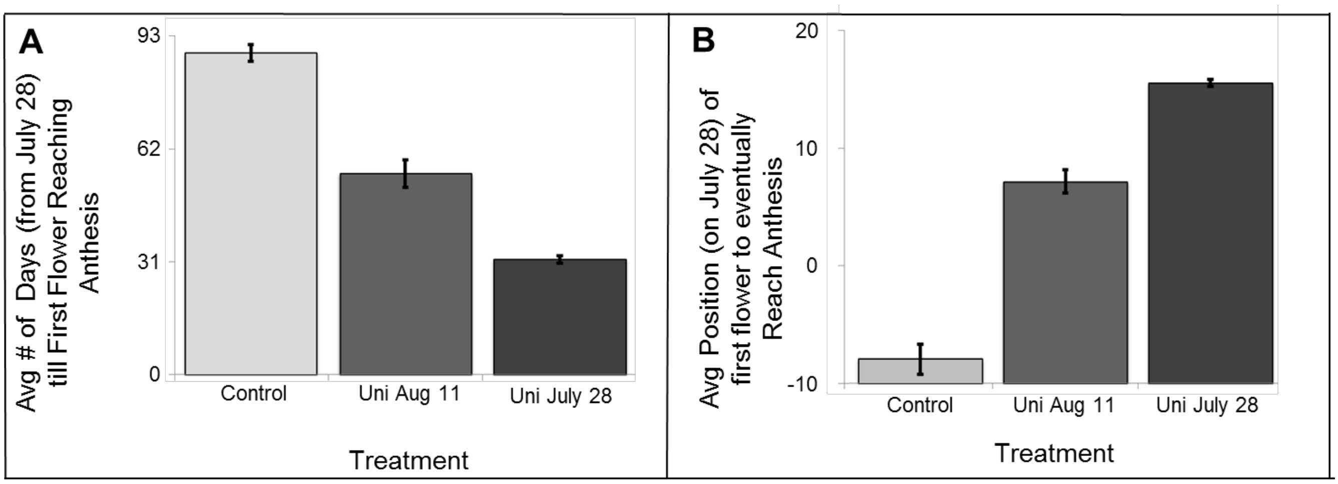

**Figure S4.** Flowers aborted due to controlled HAT conditions. (**A**) A longitudinal section viewed by light-microscopy of the most developed flower from a plant grown under LDs and HAT conditions of 34/28 °C Day/Night. (**B**) The most developed flowers of plants grown under LDs and HAT conditions of 34/22 °C. Some plants were pre-treated with 200 ppm Uni (Uni), while others were not treated (Con). Under these conditions the Uni treatment supported extra growth of the flower, yet flowers aborted before reaching anthesis. (**C–E**) Pictures taken with a stereomicroscope of a typical largest flower taken from plants grown under LDs and HAT conditions of 28/22 °C. (**C**) Complete flower in which bracts (b) continued growing, while flowers aborted after forming the gynoecium. (**D**) Flower, as in (**C**), after bract removal. (**E**) Flower as in (**D**) after sepal (s) removal. Stamens (st) and gynoecium (g) have already differentiated. The bar in A is 0.1 mm; bars in B–E are 1 mm. Notice that flowers are aborted at an earlier stage at higher temperatures. The width of the largest flower (calculated without bracts) in these pictures is 0.15 mm under 34/28 °C, 2.8 mm under 34/22 °C and 19.7 mm under 28/22 °C. Adding Uni allowed flowers of 34/22 °C plants to reach 16.3 mm before aborting.

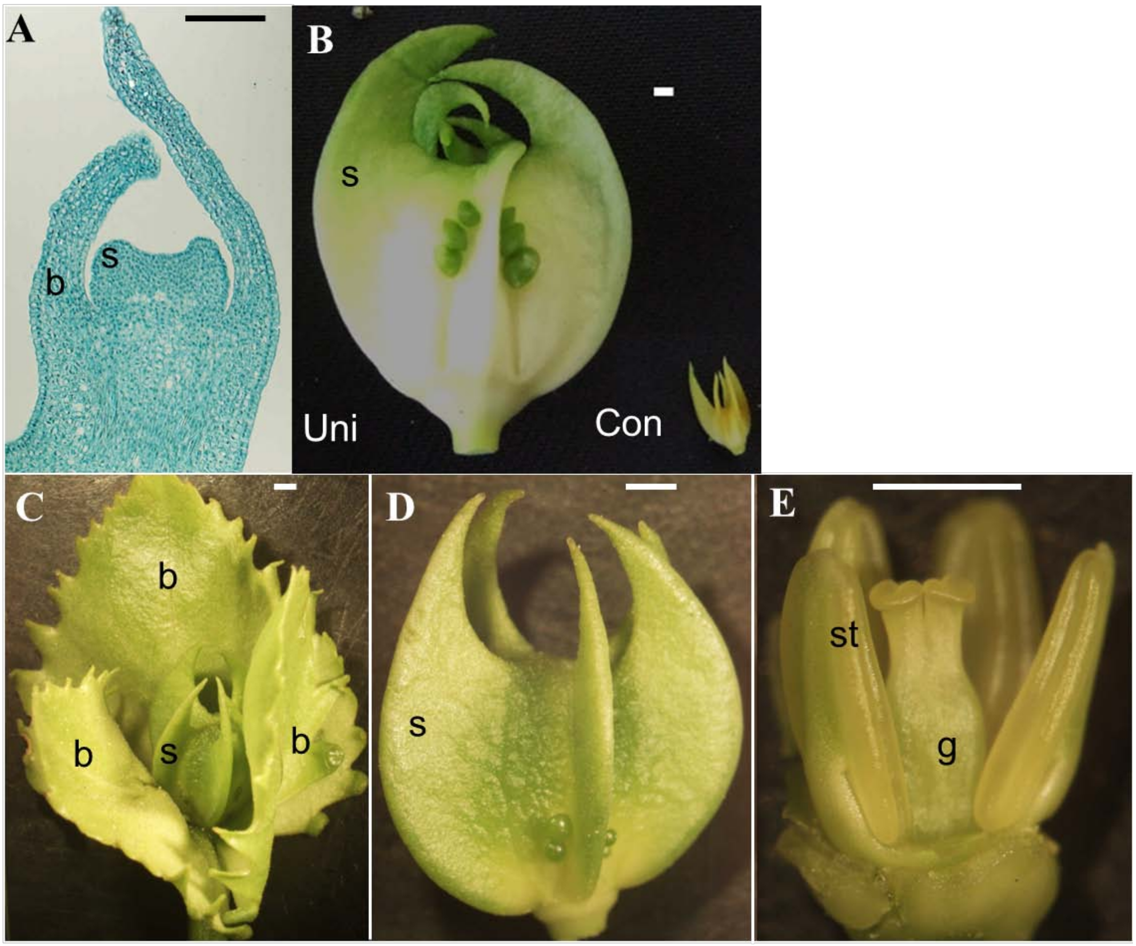


© 2014 by the authors; licensee MDPI, Basel, Switzerland. This article is an open access article distributed under the terms and conditions of the Creative Commons Attribution license (http://creativecommons.org/licenses/by/3.0/).
